# Simultaneous learning of instantaneous and time-delayed genetic interactions using novel information theoretic scoring technique

**DOI:** 10.1186/1752-0509-6-62

**Published:** 2012-06-12

**Authors:** Nizamul Morshed, Madhu Chetty, Nguyen Xuan Vinh

**Affiliations:** 1 Gippsland School of Information Technology, Faculty of Information Technology, Monash University, VIC 3842, Northways Road, Australia

## Abstract

**Background:**

Understanding gene interactions is a fundamental question in systems biology. Currently, modeling of gene regulations using the Bayesian Network (BN) formalism assumes that genes interact either instantaneously or with a certain amount of time delay. However in reality, biological regulations, both instantaneous and time-delayed, occur simultaneously. A framework that can detect and model both these two types of interactions simultaneously would represent gene regulatory networks more accurately.

**Results:**

In this paper, we introduce a framework based on the Bayesian Network (BN) formalism that can represent both instantaneous and time-delayed interactions between genes simultaneously. A novel scoring metric having firm mathematical underpinnings is also proposed that, unlike other recent methods, can score both interactions concurrently and takes into account the reality that multiple regulators can regulate a gene jointly, rather than in an isolated pair-wise manner. Further, a gene regulatory network (GRN) inference method employing an evolutionary search that makes use of the framework and the scoring metric is also presented.

**Conclusion:**

By taking into consideration the biological fact that both instantaneous and time-delayed regulations can occur among genes, our approach models gene interactions with greater accuracy. The proposed framework is efficient and can be used to infer gene networks having multiple orders of instantaneous and time-delayed regulations simultaneously. Experiments are carried out using three different synthetic networks (with three different mechanisms for generating synthetic data) as well as real life networks of *Saccharomyces cerevisiae*, *E. coli* and cyanobacteria gene expression data. The results show the effectiveness of our approach.

## Background

**Figure 1:**
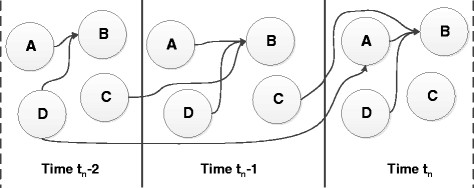
Example of network structure with both instantaneous and time-delayed interactions.

In any biological system, various genetic interactions occur concurrently amongst different genes. While some genes interact almost instantaneously, other genes could have time delayed interactions (see Figure 1). From a biological perspective, instantaneous regulations represent the scenarios where the effect of a change in the expression level of a regulator gene is carried on to the regulated gene (almost) instantaneously. In such cases, the effect is reflected almost immediately in the regulated gene’s expression level^a^. On the other hand, in cases where regulatory interactions are time-delayed, its effect will be seen on the regulated gene after a finite time delay.

Bayesian network and its extension, dynamic Bayesian network (DBN), has found significant applications in the modeling of genetic interactions [1,2]. To the best of our knowledge, prior works on inter and intra-slice connections in the dynamic probabilistic network formalism [3,4] have modelled a DBN using an initial network and a transition network employing the 1st-order Markov assumption, where the initial network exists only during the initial period of time and subsequently the dynamics is expressed using only the transition network. Realising that a *d*-th order DBN has variables replicated *d* times, a 1st-order DBN for this task^b^ is therefore usually limited to around 10 variables. Alternately, if a 2nd-order DBN model is chosen, it can mostly deal with 6-7 variables [5]. Thus, prior works on DBNs were either unable to discover these two interactions simultaneously or were unable to fully exploit its potential, thereby restricting studies to simpler network configurations. However, since our proposed approach does not replicate variables, we can study any complex network configuration without limitations on the number of nodes. Zou et al. [2], while highlighting the existence of both instantaneous and time-delayed interactions among genes while considering the parent-child relationships of a particular order, did not account for the regulatory effects of other parents (having different order of regulation than the current one) on that particular child. This is in violation of the biological reality that parents with various orders of regulation can jointly regulate a child. Our proposed method supports multiple parents to regulate a child simultaneously, with different orders of regulation. Moreover, the limitation of detecting genetic interactions like A⇔B, which are prevalent in genetic networks [6], is also overcome with the proposed method. Experiments conducted using both synthetic and real-life GRNs show the effectiveness of our approach.

## Results and discussion

We evaluate our proposed method by studying both synthetic networks and real-life biological networks of *Saccharomyces cerevisiae* (yeast), *E. coli* and cyanobacteria. The overall accuracy of the inference method and correctness of the modeling approach is evaluated by four widely accepted performance measures given below. The terms, TP, FP, TN and FN, used in the following expressions respectively mean the number of true positives, number of false positives, number of true negatives and number of false negatives. 

1. ***Sensitivity(Se):*** It measures the proportion of true connections which are correctly inferred. It is defined as follows:

(1) $$\;\text{Se}=\frac{\text{TP}}{\text{TP}+\text{FN}}$$

2. ***Specificity (Sp):*** Specificity is defined by the following equation:

(2) $$\;\text{Sp}=\frac{\text{TN}}{\text{TN}+\text{FP}}$$

3. ***Precision (Pr):*** Precision is proportional to the inferred connections which are correct. It is defined as follows:

(3) $$\;\text{Pr}=\frac{\text{TP}}{\text{TP}+\text{FP}}$$

4. ***F-score (F):*** Biologically, a good reconstruction algorithm should infer as many correct arcs as possible, in addition to the criteria that most of the inferred arcs should be correct. The F-score measure is the harmonic mean of *Se* and *Pr*[7] and represents a compromise between these two objectives:

(4) $$\;\text{F}=\frac{2\text{Pr}\;\text{Se}}{\text{Pr}+\text{Se}}$$

Since our method uses discrete data for the statistical significance tests embedded in the scoring function, we applied the Persist [8] algorithm to discretize the data into 3 levels. The confidence level (*α*) is set to 0.9. We will use a local search in the DAG space with the classical operators of arc addition, arc deletion and arc reversal. The starting point of the search is always an empty graph. The parameters for all the other methods that are used for comparison are set to their default values mentioned in their user manuals.

### Synthetic network

#### Synthetic network using differential equation based models

**Figure 2:**
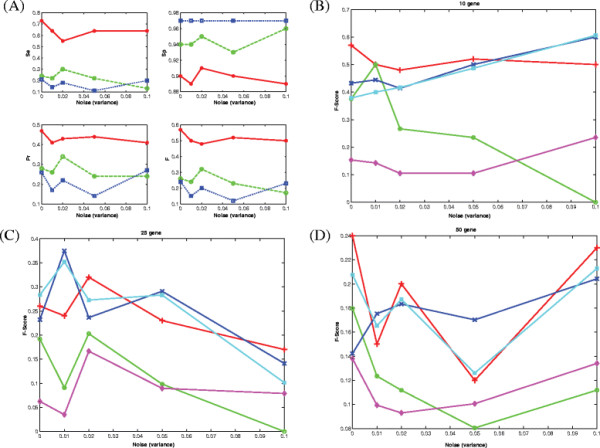
**Reconstruction of synthetic networks generated using differential equation based methods.** (**A**) How performance of our method varies with network size and noise (Red(*)-10 gene; Green(o)-25 gene; Blue(square)-50 gene). The X-axes represent the 5 levels of noise used, whereas the Y axes represent the corresponding performance measures (see text). (**B**)-(**D**) Comparison of performance with 3 other methods for the 10, 25 and 50-gene network. Red(+)-Proposed, Green(o)-BANJO, Blue(x)-BNFinder+BDe, Cyan(square)-BNFinder+MDL, Magenta(diamond)-TDARACNE. X axes-noise levels, Y axes-*F-Score*. See text for details.

For performing studies using synthetic networks, we generated 3 random networks of size 10, 25 and 50 using the genenetweaver tool [9]. This tool has been used to generate in silico benchmarks in the DREAM (both DREAM3 [10] and DREAM4 [11]) challenge initiative. The tool is able to obtain biologically plausible network topologies (and also biologically plausible network dynamics) of a given size by extracting random sub-networks of *Saccharomyces cerevisiae* and *E. Coli*[9,12]. We used the tool to generate time series data as in the DREAM4 challenge with ten different perturbations for each experiment. Initial and final timestamps for the simulations were 0 and 1000, respectively, and the time step was 50. One of the objectives of this experiment was to test the usefulness of the proposed approach in the presence of noise in mRNA expression levels. Since microarray experiments can incur a wide range of noise levels depending on the technology, environment and the subject under study, we experimented under various noise levels that are likely to be present in the expression data. To mimic a real-life noisy environment, as in [13,14], we added 5 different noise levels to the data samples (random Gaussian noise with zero mean and variance, *σ*^2^ = 0.0, 0.01, 0.02, 0.05, 0.10). The performance, measured by the four performance measures, corresponding to the three different sized networks is reported in Figure 2. Figure 2(A) shows the performance variation as a function of network size and noise level. The X-axes represent the noise levels while the Y-axes represent the corresponding performance measures (*Se, Sp, Pr, F*). In Figure 2(B)-(D), we compare our approach with three other methods, namely TDARACNE, BANJO and BNFinder (BDe and MDL) using the F-Score (results corresponding to other measures are available in Additional file 1). It is evident from the results that there is no clear winner in all the cases. Some methods perform good in some cases, while others outperform it in other cases. However, it is clear that our proposed approach, albeit not always the best, it is always among the top performers and has consistently superior performance.

#### Probabilistic network of yeast

**Figure 3:**
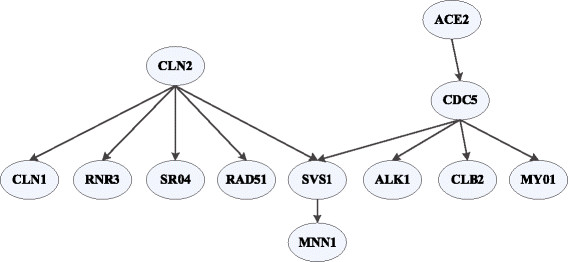
**Yeast cell cycle sub-network**[15].

We use a sub-network from the yeast cell cycle, shown in Figure 3, taken from Husmeier et al. [15]. The network consists of 12 genes and 11 interactions. For each interaction, we randomly assigned a regulation order of 0, 1, 2 or 3. We used two different conditional probabilities for the interactions between the genes, namely, the noisy regulation according to a binomial distribution and the noisy XOR-style co-regulation. For the binomial distribution dependent noisy regulation, the parameters were set as follows: excitation: P(on|on) = 0.9, P(on|off) = 0.1; inhibition: P(on|on) = 0.1, P(on|off) = 0.9. For the noisy XOR-style co-regulation the parameters were set as: P(on|on, on) = P(on|off, off) = 0.1, P(on|on, off) = P(on|off, on) = 0.9 [15]. Eight confounder nodes were also added, resulting in the total number of nodes to be 20.

We used 30, 50 and 100 samples, generated 5 datasets in each case and compared our approach with two other DBN based methods, namely BANJO [16] and BNFinder [17]. Since these methods detect only regulations of order 1, while calculating performance measures for these methods, we ignored the exact orders for the time-delayed interactions in the target network. We could not apply TDARACNE [7] to this network since the generated data has two levels of discrete values and TDARACNE returns error when applied to such discrete datasets. We show the results for this network in Table 1, where we observe that our method, coupled with a high precision, outperforms the other two in terms of both sensitivity and specificity. The F-score is also the best in all the cases. This points to the strength of our method in discovering complex interaction scenarios where multiple regulators may jointly regulate target genes with varying time-delays.

**Table 1:** Comparison of proposed method with BANJO and BNFinder on the yeast sub-network

	**N=30**				**N=50**				**N=100**			
	***Se***	***Sp***	***Pr***	***F***	***Se***	***Sp***	***Pr***	***F***	***Se***	***Sp***	***Pr***	***F***
**Proposed**	**0.62±**	0.992±	0.57±	0.59±	**0.80±**	**1.0±**	**0.79±**	**0.79±**	**0.82±**	**1.0±**	**0.76±**	**0.79±**
**Method**	**0.12**	0.0045	0.11	0.11	**0.04**	**0.0**	**0.07**	**0.05**	**0.06**	**0.0**	**0.03**	**0.04**
**BNFinder**	0.53±	**0.996±**	**0.68±**	**0.59±**	0.62±	0.997±	0.74±	0.67±	0.69±	0.997±	0.74±	0.72±
**+BDe**	0.04	**0.0006**	**0.02**	**0.02**	0.04	0.0019	0.13	0.06	0.08	0.0007	0.06	0.07
**BNFinder**	0.51±	**0.996±**	0.63±	0.56±	0.60±	0.996±	0.68±	0.63±	0.65±	0.996±	0.69±	0.67±
**+MDL**	0.08	**0.0006**	0.07	0.08	0.05	0.0022	0.15	0.09	0.0	0.0	0.04	0.02
**BANJO**	0.51±	0.987±	0.49±	0.46±	0.55±	0.993±	0.57±	0.55±	0.60±	0.995±	0.61±	0.61±
	0.08	0.01	0.2	0.15	0.09	0.0049	0.23	0.16	0.08	0.0014	0.09	0.08

#### Synthetic network of glucose homeostasis

In higher eukaryotes, glucose homeostasis is maintained via a complex system involving many organs and signaling mechanisms. The liver plays a crucial role in this system by storing glucose as glycogen when blood glucose levels are high, and releasing glucose into the bloodstream when blood glucose levels are low. To accomplish its task, the liver responds to circulating levels of hormones, mainly insulin, epinephrine, glucagon, and glucocorticoids [18].

**Figure 4:**
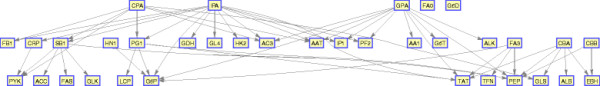
Synthetic network of glucose homeostasis.

Le et al. [18] conducted an extensive review of the literature regarding the biological components affecting perinatal glucose metabolism. Based on the study, a Bayesian Network model of glucose homeostasis containing 35 nodes and 52 interactions (shown in Figure 4) was constructed. We used the model for generating datasets of varying size (50, 75 and 100 samples), having first and second-order regulations using the Bayes Net Toolbox [19]. The random multinomial CPDs used by this approach of data generation were obtained by sampling from a Dirichlet distribution with hyper-parameters chosen by the method^c^ described in [20] with a corresponding Equivalent Sample Size (ESS) value of 10. The choice of this prior distribution for the conditional parameters ensures a reasonable level of dependence between d-connected variables in the generative structure [20].

We compare our method with the three other methods that were used previously for comparison, namely BANJO [16] and BNFinder [17](using BDe and MDL). While calculating performance measures for these methods, we ignored the exact orders for the time-delayed interactions in the target network. Similar to the probabilistic network of yeast, we could not apply TDARACNE for this network due to error occurring because TDARACNE is unable to cope with the discrete data. The results are shown in Table 2. We observe that, both in terms of specificity and precision, our method outperforms others. The F-score is the highest in all the cases, indicating a good balance between sensitivity and precision.

**Table 2:** Comparison of proposed method with BANJO and BNFinder on the glucose homeostasis network

	**N=50**				**N=75**				**N=100**			
	***Se***	***Sp***	***Pr***	***F***	***Se***	***Sp***	***Pr***	***F***	***Se***	***Sp***	***Pr***	***F***
**Proposed**	0.50	**0.9812**	**0.54**	**0.52**	0.46	**0.9914**	**0.71**	**0.56**	0.54	**0.9906**	**0.72**	**0.62**
**Method**												
**BNFinder**	0.48	0.9488	0.29	0.37	0.52	0.9506	0.32	0.39	**0.56**	0.9557	0.36	0.44
**+BDe**												
**BNFinder**	**0.54**	0.948	0.31	0.40	**0.56**	0.9395	0.29	0.38	0.54	0.9369	0.27	0.37
**+MDL**												
**BANJO**	0.52	0.97	0.44	0.47	0.48	0.9838	0.57	0.52	0.54	0.9881	0.67	0.60

### Real-life biological data of *saccharomyces cerevisiae* (IRMA)

To validate our method with a real-life biological gene regulatory network, we investigate a recent network reported in [21]. In that significant work, the authors built a network, called IRMA, of the yeast *Saccharomyces cerevisiae*[21]. They tested the transcription of network genes by culturing the cells in presence of galactose and glucose. The network is composed of five genes regulating each other; it is also negligibly affected by endogenous genes. It is one of the first attempts at building a reference data set having an accurately known target network [7]. There are two sets of gene profiles called Switch ON and Switch OFF for this network, each containing 16 and 21 time series data points, respectively. A ’simplified’ network, ignoring some internal protein level interactions, is also reported in [21]. To compare our reconstruction method, we consider 3 other methods, namely, TDARACNE [7], BANJO [16] and BNFinder [17].

#### IRMA ON dataset

The performance comparison amongst various method based on the ON dataset is shown in Table 3. We observe that our method clearly outperforms the others. There are no false predictions and precision is highest. The sensitivity and F-score measures are also very high.

**Table 3:** Performance comparison based on IRMA ON dataset

	**Original Network**				**Simplified Network**			
	***Se***	***Sp***	***Pr***	***F***	***Se***	***Sp***	***Pr***	***F***
**Proposed Method**	**0.63**	**1.0**	**1.0**	**0.77**	**0.67**	**1.0**	**1.0**	**0.80**
**TDARACNE**	**0.63**	0.88	0.71	0.67	**0.67**	0.90	0.80	0.73
**BNFinder+BDe**	0.13	0.82	0.25	0.17	0.17	0.80	0.33	0.22
**BNFinder+MDL**	0.13	0.82	0.25	0.17	0.17	0.80	0.33	0.22
**BANJO**	0.25	0.76	0.33	0.27	0.50	0.70	0.50	0.50

#### IRMA OFF dataset

Due to the lack of ’stimulus’, it is relatively difficult to reconstruct the exact network from the OFF dataset [7]. As a result, the overall performances of all the algorithms suffer to some extent. The comparison is shown in Table 4. Again, we observe that our method reconstructs the gene network with high precision. Specificity is also quite high, implying that the inference of false positives is low.

**Table 4:** Comparison based on IRMA OFF dataset

	**Original Network**				**Simplified Network**			
	***Se***	***Sp***	***Pr***	***F***	***Se***	***Sp***	***Pr***	***F***
**Proposed Method**	0.50	**0.94**	**0.80**	**0.62**	0.50	**0.90**	**0.75**	**0.60**
**TDARACNE**	**0.60**	-	0.37	0.46	**0.75**	-	0.50	**0.60**
**BNFinder+BDe**	0.13	0.82	0.25	0.17	0.33	0.80	0.50	0.40
**BNFinder+MDL**	0.13	0.82	0.25	0.17	0.33	0.80	0.50	0.40
**BANJO**	0.38	0.88	0.60	0.46	0.33	**0.90**	0.67	0.44

### Yeast KEGG pathway reconstruction

In order to test the proposed method’s performance on yeast *S. cerevisiae* cell cycle, we selected a eleven gene network of the G1-phase: Cln3, Cdc28, Mbp1, Swi4, Clb6, Cdc6, Sic1, Swi6, Cln1, Cln2, Clb5. The data used was obtained from the *cdc28* experiment of Spellman et al. [22]. In the later stage of the G1-phase, the Cln3-Cdc28 protein kinase complex activates two transcription factors, MBF and SBF, and these promote the transcription of some genes important for budding and DNA synthesis [7,23]. Entry into the S-phase requires the activation of the protein kinase Cdc28p through binding with Clb5 or Clb6, and also the destruction of Sic1 [24]. Also, Swi4 becomes associated with Swi6 to form the SCB complex that activates CLN1 and CLN2 in late G1. Mbp1 forms the MCB-binding factor complex with Swi6, which activates DNA synthesis genes and S-phase cyclin genes CLB5 and CLB6 in late G1 [7]. In budding yeast, commitment to DNA replication during the normal cell cycle requires degradation of the cyclin-dependent kinase (CDK) inhibitor Sic1. The G1 cyclin-CDK complexes Cln1-Cdk1 and Cln2-Cdk1 initiate the process of Sic1 removal by directly catalyzing Sic1 phosphorylation at multiple sites [7,25].

**Figure 5:**
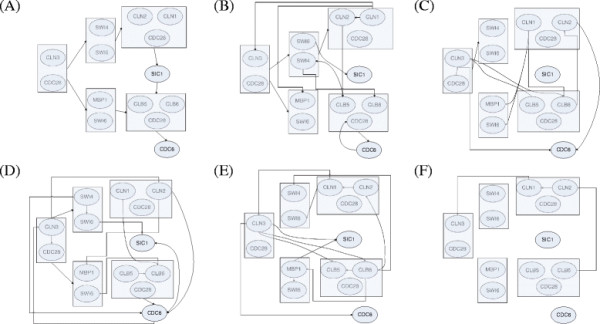
**Reconstruction of Yeast KEGG Pathway **[26]. (**A**) Target Network. (**B**) Network Inferred by proposed approach. (**C**) Network Inferred by TDARACNE. (**D**) Network Inferred by BANJO. (**E)**Network Inferred by BNFinder+BDe. (**F**) Network Inferred by BNFinder+MDL.

In Figure 5(B)-(F), we report network graphs reconstructed by our proposed approach, TDARACNE, BNFinder(BDe and MDL) and BANJO. We also report the KEGG pathway [26] of the cell-cycle in yeast in 5(A). Since the ground truth for this network is not known, instead of applying performance measures as a means of determining network accuracy, we refer to the available correct interactions obtained from the KEGG pathway [26] and identify which of the predicted interactions are correct or otherwise. We observe from the results that our approach correctly identifies the regulation of SWI4-SWI6 and MBP1-SWI6 complex by the CLN3-CDC28 complex. Also, the proposed approach infers that the SWI4-SWI6 complex regulates the CLN1-CLN2-CDC28 complex, which is correct. Two more interactions inferred by our approach (CLN1→CLN2 and CLB5-CLB6-CDC28→CDC6) are also correct based on the KEGG pathway. Overall we observe that none of the methods perform particularly well on this network. However, the number of correct predictions by our method (5) is higher than the other methods.

### SOS DNA repair network of *E. coli*

**Figure 6:**
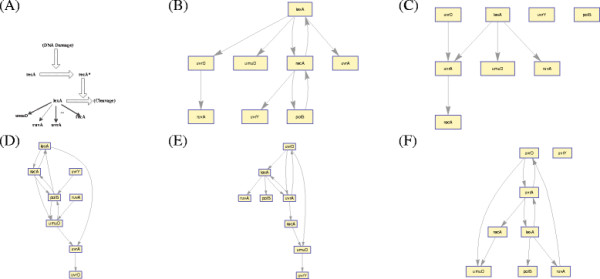
**Reconstruction of SOS DNA Repair Network (A) Target Network.** (**B**) Network Inferred by proposed approach. (**C**) Network Inferred by TDARACNE. (**D**) Network Inferred by BANJO. (**E**) Network Inferred by BNFinder+BDe. (**F**) Network Inferred by BNFinder+MDL.

We analyze the well-known SOS DNA repair network in *E. coli* as shown in Figure 6(A). This GRN is well known for its responsibility of repairing the DNA if it gets damaged. It is the largest, most complex, and best understood DNA damage-inducible network to be characterized to date.

The expression of the genes in the SOS regulatory network is controlled by a complex circuitry which involves the RecA and LexA proteins [27]. Normally LexA acts as the master repressor of more than 20 genes, including lexA and recA genes. This repression is done by its binding to the interaction sites in the promoter regions of these genes. When DNA damage occurs, one of the SOS proteins, RecA, acts as a sensor. By binding to single-stranded DNA, it becomes activated, senses the damage and mediates LexA autocleavage [27]. The drop in LexA levels in turn stops the repression of the SOS genes and activates them. When the damage has been repaired, the level of activated RecA drops and it stops mediating LexA autocleavage. LexA level in turn increases, starting repression of the SOS genes, and the cell then returns to its normal state.

The expression data sets of the SOS DNA repair system were obtained from Uri Alon Lab [28]. These data are expression kinetics of 8 genes namely uvrD, lexA, umuD, recA, uvrA, uvrY, ruvA and polB. Four experiments were done for various UV light intensities (Exp. 1 and 2:5*Jm*^−2^, Exp. 3 and 4:20*Jm*^−2^). In each experiment, the above 8 genes were monitored at 50 instants which are evenly spaced by 6 minutes intervals.

The results corresponding to Experiment 1 is presented in Figure 6(B). Along with our result, we include the results from BANJO, TDARACNE and BNFinder in Figure 6(C)-(F) and the target network in 6(A). The results corresponding to the other experiments are available in Additional file 2, Additional file 3, Additional file 4, Additional file 5 and Additional file 6. From the results, we observe that our method correctly identifies lexA and recA as the ’hub’ genes for this network. Again, the exact ground truth for this network is not precisely known, and hence it is not possible to calculate the well-known performance measures. Instead, using the known interactions obtained from literature [13,14], an analysis of correct and incorrect predictions by our method is obtained and shown in Table 5. We observe that most of the interactions inferred by our proposed method are correct. It successfully infers lexA as the regulator of uvrA, uvrD, umuD and recA. Also, considering the indirect regulation of RecA through LexA, two more interactions, namely recA→uvrY and recA→polB can also be considered correct. In contrast, 3 of the 5 identified interactions by TDARACNE [7] are correct. Most of the interactions identified by BANJO and BNFinder+MDL are incorrect. BNFinder+BDe successfully identifies regulation of ruvA, polB and uvrA by lexA. In addition, the regulation of umuD by recA can also be considered correct. However, compared to these methods, our proposed method infers the highest number of correct predictions. Number of incorrect predictions is also very low for our method.

**Table 5:** Analysis of individual interactions inferred by proposed method

		**correct/**
**Regulator**	**Target**	**incorrect**
	uvrD	correct
	umuD	correct
LexA		
	recA	correct
	uvrA	correct
	uvrY	correct^a^
RecA		
	polB	correct^a^
uvrD	ruvA	incorrect

^a^correct considering indirect regulation of RecA through LexA

### Network analysis of strongly cycling genes in cyanobacteria, *Cyanothece* sp. ATCC 51142

**Figure 7:**
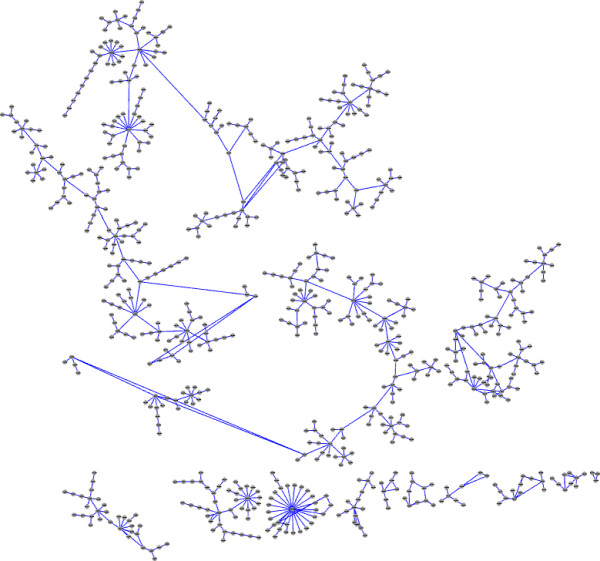
Network inferred by proposed approach.

**Figure 8:**
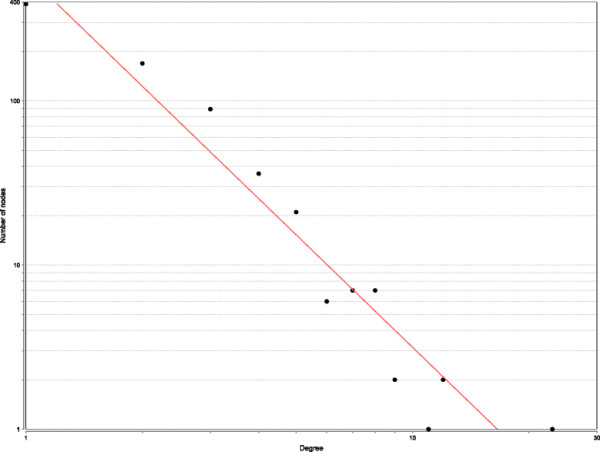
**Degree distribution analysis of the resultant network of *cyanothece*.** We used a power law fit which yields *R*^2^ = 0.93. The result confirms that the inferred network is scale-free.

To study our approach on a large scale network, we use a network of a strain of cyanobacteria, namely *Cyanothece* sp. strain ATCC 51142 [29]. The microarray data corresponding to the genes were collected from two publicly available genome-wide microarray data sets of *Cyanothece*, performed in alternating light-dark (LD) cycles with samples collected every 4h over a 48h period: the first one starting with 1h into dark period followed by two DL cycles (DLDL), and the second one starting with two hours into light period, followed by one LD and one continuous LL cycle (LDLL) [30]. In total, there were 24 samples. Using a threshold filter with a 2-fold change cutoff, 730 genes were selected for the analysis. The genes are responsible for performing the major tasks of energy metabolism and respiration, nitrogen fixation, protein translation and folding, and photosynthesis, along with several other tasks. Result obtained using our method is shown in Figure 7. The degree distribution is shown in Figure 8. To compare our result with the other methods, we applied BANJO, BNFinder(BDe and MDL) and TDARACNE. The results of all the three except BNFinder(BDe) was not satisfactory. As a result, we compare our method only with BNFinder+BDe.

Similar to other large scale datasets (e.g. the Human HeLa cell data [31], the *Arabidopsis L. Heynth* dataset [32]), the microarray data set for cyanobacteria also has very few samples. Moreover, being not a well-studied organism, it requires caution in the interpretation of results. We note that GRN reconstruction studies of cyanobacteria reported earlier (e.g. [29,33,34]) commonly emphasise an evaluation criteria, namely “functional enrichment” analysis of sub-networks. Further, another common feature noted for genetic networks [35-37] is that transcriptional regulatory networks possess the scale free nature of the network topology^d^. Since we have limited samples and also because the ground truth is unknown, we have carried out the evaluation of the inferred network using both: (i) statistical means i.e. GO functional enrichment analysis (using both *p* = 0.05 and *p* = 0.10), and (ii) the *R*^2^ measure of the power-law fit of the network to establish its scale-free nature.

The enrichment analysis was done by using gene ontology (GO) database (compiled using two sources: one from the Cyanobase database [38], and another from genome-wide amino sequence matching using the Blast2GO software suite [39]; the the compiled database is available in Additional file 7), where every GO terms appearing in each sub-network is assessed to find out whether a certain functional category is significantly over-represented in a certain sub-network/cluster, more than what would be expected by chance. The Cytoscape [40] plugin BiNGO [41] was used for GO functional category enrichment analysis. For BiNGO, we use the combined and filtered gene set as the reference set, the hypergeometric test as the test for functional over-representation, and False Discovery Rate (FDR) as the multiple hypothesis testing correction scheme.

First, we present the results corresponding to *p* = 0.05. The network obtained by BNFinder+BDe has 16 sub-networks each containing at least 3 genes. Of these, 6 sub-networks have significantly enriched functionalities (as determined by the GO functional enrichment test). Of the other 10, we compute the 3 most densely connected hubs for each sub-network, and in 2 of 10 such sub-networks, the hubs have defined significantly enriched functionalities. On the other hand, in our result, there are 14 sub-networks in total having at least 3 genes. Of these, 3 sub-networks have defined enriched functions (the largest sub-network has the role of nitrogen fixation according to the enrichment test). Of the other 11, we compute the 3 most densely connected hubs for each sub-network, and in 5 of the 11 such sub-networks, the hubs have defined significantly enriched functionalities.

The results corresponding to *p* = 0.10 show that for BNFinder+BDe, 7 sub-networks have enriched functionalities (as determined by the test). Of the other 9, we compute the 3 most densely connected hubs for each sub-network, and in 2 of the 9 such sub-networks, the hubs have defined enriched functionalities. On the contrary, the result using our approach has 5 sub-networks with defined significantly enriched functions (the largest sub-network has the role of nitrogen fixation, similar to the *p* = 0.05 case). Of the other 9, we compute the 3 most densely connected hubs for each sub-network, and in 6 of the 9 such sub-networks, the hubs have defined significantly enriched functionalities.

We also test the networks to assess whether they are scale free, using a power law fit. The *R*^2^ value of the fit corresponding to our network is 0.93, which is a better fit compared to BNFinder+BDe (0.62).

## Conclusion

In this paper, we propose a framework that can simultaneously represent instantaneous and time-delayed genetic interactions. The proposed scoring metric uses information theoretic quantities having not only relevant properties but also implicitly includes the biological truth that some genes may jointly regulate other genes. Incorporating these novel features, we have implemented a *score+search* based GRN reconstruction algorithm. Experiments have been performed on different synthetic networks of varying complexities and also on real-life biological networks. Our method shows improved performance compared to other recent methods, both in terms of reconstruction accuracy and number of false predictions and at the same time maintaining comparable or better true predictions. A natural extension of the described method can be incorporation of a-priori knowledge from sources like protein-protein interactions databases and fusing the knowledge with existing regulatory networks to make the inferred networks much more reliable, and we are pursuing this objective. Along with these extensions, the proposed approach would improve the accuracy of gene regulatory network reconstruction and enhance research in systems biology.

## Methods

### The representational framework

Let us model a gene network containing *n* genes (denoted by $X_{1},X_{2}\ldots ,X_{n}$) with a corresponding microarray dataset having *N* time points. A basic DBN-based GRN reconstruction method would try to find associations between genes *X*_*i*_ and *X*_*j*_ by taking into consideration the data $x_{i1},\ldots ,x_{i(N-\delta )}$ and $x_{j(1+\delta )},\ldots ,x_{\text{jN}}$ or vice versa (small case letters mean data values in the microarray), where 1 ≤ *δ* ≤ *d*. That is, it will take into consideration the *d*-th order Markov rule, for a gene having a maximum order of regulation *d* with its parents. This will effectively enable this model to capture at most *d*-step time delayed interactions. Conversely, a basic BN-based strategy would use the entire set of *N* time points and it will capture regulations that are effective instantaneously.

**Figure 9:**
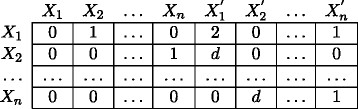
The adjacency matrix based approach for the representation.

Now, to represent both instantaneous and multiple step time-delayed interactions, we consider an adjacency matrix based structure as shown in Figure 9. The zero entries in the figure denote no regulation. For the first *n* columns, the entries marked by 1 correspond to instantaneous regulations whereas for the last *n* columns non-zero entries denote the order of regulation. As an example, the entry 1 in the cell (*X*_1_, *X*_2_) means *X*_1_ has (almost) instantaneous regulatory effect on *X*_2_. Similarly, the entry *d* in the cell $\left(X_{n},X_{2}^{{}^{\prime }}\right)$ means *X*_*n*_ regulates *X*_2_ with a *d*-step time delay. Using this representation, we do not need to replicate layers of interactions for each increment in the order of regulations, making it efficient and particularly suitable for representing GRNs, where higher-order regulations is a common phenomenon.

Complications in the alignment of data samples can arise if the parents have different orders of regulation with the child node. To make this notion clear, we describe an example where we have already assessed the degree of interest in adding two parents (gene *B* and *C*, having third and first order regulations, respectively) to the gene under consideration, *X*. Now, we want to assess the degree of interest in adding gene *A* as a parent of *X* with a second order regulatory relationship, that is we want to compute^e^*MI*(*X*, *A*^2^|{*B*^3^, *C*^1^}), where superscripts on the parent variables denote the order of regulation it has with the child node.

**Figure 10:**
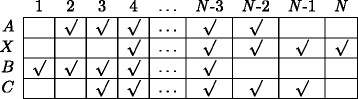
Sample points used for the calculation of the Mutual Information (MI).

There are two possibilities to consider. The first one corresponds to the scenario where the time-series data is not periodic. In this case, we cannot use all the *N* samples for MI computation, rather we have to use (*N* − *δ*) samples where *δ* is the maximum order of regulation that the gene under consideration has, with its parent nodes (3 in this example). Figure 10 shows how the alignment of the samples can be done for the current example. In the figure, we have *N* samples and since *δ* = 3, we can effectively use (*N* − 3) samples.

The *√*symbol inside a cell denotes that this data sample will be used for MI computation, whereas empty cells denote that these data samples will not be considered for computing the MI. Similar alignments will need to be done for the other case, where the data is considered to be periodic (e.g., datasets of yeast compiled by [42] show such cyclic behavior [43]). However, we can use all the *N* data samples in this case, where the data is shifted in a circular manner.

The interpretation of the results obtained from an algorithm that uses this framework can be done in a straightforward manner. Using this framework and the aligned data samples, if we construct a network where we observe, for example, arc $X_{1}\to X_{n}^{{}^{\prime }}$ having order *δ*, we conclude that the inter-slice arc between *X*_1_ and *X*_n_ is inferred and *X*_1_ regulates *X*_*n*_ with a *δ*-step time-delay. Similarly, if we find an arc *X*_2_ → *X*_*n*_, we say that the intra-slice arc between *X*_2_ and *X*_n_ is inferred and a change in the expression level of *X*_2_ will almost immediately effect the expression level of *X*_n_. To ensure consistency in the resulting Bayesian networks, the following 3 conditions must also be satisfied: 

1. The network must be a directed acyclic graph.

2. The inter-slice arcs must go in the correct direction (no backward arc).

3. Interactions remain existent independent of time (stationarity assumption).

### Our proposed scoring metric, CCIT

We share the same idea with MIT (Mutual Information Tests) [44] and MDL (the Minimum Description Length principle) for developing a scoring metric that can score both instantaneous and time-delayed interactions simultaneously: to use the MI/log-likelihood measure between a node *X*, and its parents, *Pa(X)* for measuring the degree of association between them, and penalizing structural complexity. The first part aims at minimizing the Kullback-Leibler (KL) divergence between the joint distribution corresponding to the original network (*p*_*D*_) and the graph under consideration (*p*_*G*_), according to the following equation:

(5) $$\;\underset{G\in G_{n}}{\text{argmin}}\;\text{KL}(p_{D},p_{G})=\underset{G\in G_{n}}{\text{argmax}}\;\overset{n}{\underset{\underset{Pa_{G}(X_{i})\neq \phi }{i=1}}{\sum }}\;\text{MI}(X_{i},Pa_{G}(X_{i}))$$

which is equivalent to maximizing the log-likelihood (i.e., the higher the MI/log-likelihood score, the better is the network) [44]. In our approach, calculation of the MI/log-likelihood score is done in a manner which is similar to the approaches in MIT/MDL, with a major difference: calculation of score (using MI/log-likelihood) in case of joint regulation. To make the notion clear consider Figure 1. Using MIT, the MI part for scoring for gene *B* is^f^*MI*(*B*, {*A*^0^, *D*^0^}) + *MI*(*B*, *C*^1^) (similar calculations of log-likelihood will be used for MDL). As we can see, the calculation of MI/log-likelihood for the zero-order interactions do not take into account the parents who regulate it with time-delay. Unlike the approach in basic MIT and other approaches where zero and higher-order interactions are scored separately and then combined, in our approach, we also condition (during computation) on parents which have different orders of regulation with the target gene. The marginal probability for each node of this model thus becomes:

(6) $$\;P(X[t]|X[t-1],\ldots ,X[t-d])=\overset{n}{\underset{i=1}{\prod }}P(X_{i}[t]|\text{Pa}(X_{i}[t]))$$

The term *Pa*(*X*_*i*_[*t*]) in the above equation represents the parents of gene *X*_*i*_ at time *t*, which can be in the same time-slice or in one of the *d* previous time-slices (*d* is the maximum order of regulation) of gene *X*_*i*_ at time *t*. Thus, using our approach, the scoring function for *B* will calculate $\text{MI}(B,\{A^{0},D^{0}\}\cup \{C^{1}\})$. Scoring in this manner enables us to score both intra and inter-slice interactions simultaneously, rather than considering these two types of interactions in an isolated manner, making it specially suitable for problems like reconstructing GRNs, where occurrence of joint regulation is a common phenomenon.

The idea of penalizing complex structures is ubiquitous, finding its place in most of the scores like BIC, MIT and MDL. The penalization component for BIC and MDL are global, whereas for MIT it is specific for each variable and its parents. Being local in nature, the MIT scheme usually outperforms the other two [44]. In this scheme, the localised penalization is based on a theorem of Kullback [45], which says that for a particular confidence level *α*, the quantity 2*N.MI*(*X*_*i*_, *X*_*j*_|*Pa*(*X*_*i*_)) − *χα*,*l*_*ij*_ represents a statistical test of conditional independence, where *l*_*ij*_ is the degrees of freedom of a chi-squared distribution, and $\chi_{\alpha ,l_{\text{ij}}}$ is the statistical significance threshold. The more positive the value is, the more likely is that *X*_*i*_ and *X*_*k*_ are related (given the current parent set, *Pa*(*X*_*i*_)) and vice-versa. Thus, adding up the MI quantities for all the genes (multiplied by 2*number of samples) and subtracting the corresponding local penalization measures effectively constitute a series of conditional independence (CI) tests, and this scheme is used for scoring using MIT.

However, porting this idea of local penalization directly to a gene regulatory network which is cursed with dimensionality (there are a large number of variables (genes), but only a few samples are available), has the problem of over-penalization. This can be exemplified using Figure 1. The penalization component for gene *B* according to MIT, will be: *χ*_*α*,4_ + *χ*_*α*,12_ + *χ*_*α*,36_, assuming the special case where we have 3 levels of discrete data (the details of how these penalization components can be computed will be shown later). For a Bayesian network design having thousands of samples available, this penalization is not a problem. However, but for GRN reconstruction with samples ranging between 20-50, this penalization is too high. To remedy this situation, we propose to apply the penalization only on a per-order of regulation basis. Using this modified scheme, the penalization will be 2*χ*_*α*,4_ + *χ*_*α*,12_, which constitutes considerable savings, thereby increasing better prediction ratio (in terms of sensitivity and specificity).

The approaches described above are summarised as a scoring metric, named CCIT (Combined Conditional Independence Tests) in Equation 7. The score, when applied to a graph *G* containing *n* genes (denoted by $X_{1},X_{2}\ldots ,X_{n}$), with a corresponding microarray dataset *D*, can be expressed as:

(7) $$\;\begin{array}{lll} S_{\text{CCIT}}\left(G:D\right)=\overset{n}{\underset{\underset{\text{Pa}(X_{i})\neq \phi }{i=1}}{\sum }} & \left\{2N_{\delta_{i}}\text{.MI}(X_{i},\text{Pa}(X_{i}))\right)\; & \;\\ & \left(-\overset{\delta_{i}}{\underset{k=0}{\sum }}\;(\underset{\sigma_{i}^{k}}{\text{max}}\overset{s_{i}^{k}}{\underset{j=1}{\sum }}\chi_{\alpha ,l_{i}\sigma_{i}^{k}(j)}),\;\right\}\; \end{array}$$

Here $s_{i}^{k}$ denotes the number of parents of gene *X*_*i*_ having a *k* step time-delayed regulation and *δ*_*i*_ is the maximum time-delay that gene *X*_*i*_ has with its parents. The parent set of gene *X*_*i*_, *Pa*(*X*_*i*_) is the union of the parent sets of *X*_*i*_ having zero time-delay (denoted by $Pa^{0}(X_{i})$), single-step time-delay (denoted by $Pa^{1}(X_{i})$) and up to parents having the maximum time-delay (*δ*_*i*_). This is defined as follows:

(8) $$\;\text{Pa}(X_{i})=Pa^{0}\left(X_{i}\right)\cup Pa^{1}\left(X_{i}\right)\;\cdots \cup Pa^{\delta_{i}}\left(X_{i}\right)$$

The number of *effective* data points, $N_{\delta_{i}}$, depends on whether the data can be considered to be showing periodic behavior or not (e.g., datasets from [42] can be considered as showing periodic behavior [43]). In the case of aperiodicity, $N_{\delta_{i}}$ is determined by subtracting, from the total length of the time profile (*N*), the maximum order of the time-delay that the gene under consideration has with its parents (*δ*_*i*_).

(9) $$\;\begin{array}{c} N_{\delta_{i}}=\left\{\begin{array}{c} \;N\;\text{if data is periodic}\\ N-\delta_{i}\;\;\text{otherwise} \end{array}\right) \end{array}$$

Finally, $\sigma_{i}^{k}=(\sigma_{i}^{k}(1),\ldots ,\sigma_{i}^{k}(s_{i}^{k}))$ denote any permutation of the index set $\left(1,\ldots ,s_{i}^{k}\right)$ of the variables $Pa^{k}(X_{i})$ and $l_{i\sigma_{i}^{k}(j)}$, the degrees of freedom, is defined as follows:

(10) $$\;l_{i\sigma_{i}^{k}(j)}=\left\{\begin{array}{c} (r_{i}-1)(r_{\sigma_{i}^{k}(j)}-1)\prod_{m=1}^{j-1}r_{\sigma_{i}^{k}(m)},\text{for}2\leq j\leq s_{i}^{k}\\ \;\;\;\;\;\;(r_{i}-1)(r_{\sigma_{i}^{k}(1)}-1),\text{for}j=1 \end{array}\right)$$

where *r*_*p*_ denotes the number of possible values that gene *X*_*p*_ can take (after discretization, if the data is continuous). If the number of possible values that the genes can take is not the same for all the genes, the quantity $\sigma_{i}^{k}$ denotes the permutation of the parent set $Pa^{k}(X_{i})$ where the first parent gene has the highest number of possible values, the second gene has the second highest number of possible values and so on.

### Some properties of CCIT Score

In this section we study several useful properties of the proposed scoring metric. The first among these is the decomposability property, which is especially useful for local search algorithms:

#### Proposition 1

CCIT is a decomposable scoring metric.

#### Proof

This result is evident as the scoring function is, by definition, a sum of local scores. □

Next, we show in Theorem 1 that CCIT takes joint regulation into account while scoring and it is different than three related approaches, namely MIT [44] applied to: a Bayesian Network (which we call *MIT*_0_); a dynamic Bayesian Network (called *MIT*_1_); and also a naive combination of these two, where the intra and inter-slice networks are scored independently (called *MIT*_0 + 1_). For this, we make use of the decomposition property of MI, defined next:

#### Property 1

(Decomposition Property of MI) In a BN, if *Pa*(*X*_*i*_) is the parent set of a node *X*_*i*_, and the cardinality of the set is *s*_*i*_, the following identity holds [44]:

(11) $$\;\begin{array}{l} \text{MI}(X_{i},\text{Pa}\left(X_{i})\right)=\text{MI}\left(X_{i},X_{i1}\right)\\ \;+\overset{s_{i}}{\underset{j=2}{\sum }}\text{MI}\left(X_{i},X_{\text{ij}}|\left\{X_{i1},\ldots ,X_{i(j-1)}\right\}\right)\; \end{array}$$

#### Theorem 1

CCIT scores intra and inter-slice arcs concurrently, and is different from *MIT*_0_, *MIT*_1_ and *MIT*_0 + 1_ since it takes into account the fact that multiple regulators may regulate a gene simultaneously, rather than in an isolated manner.

#### Proof

**Figure 11:**
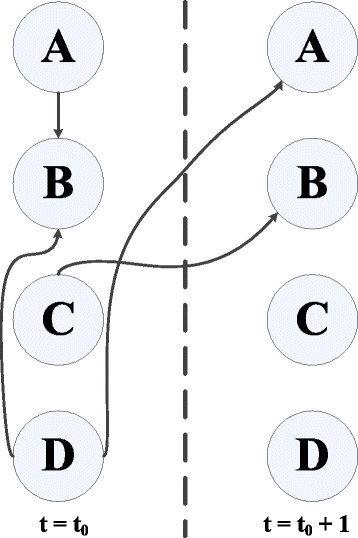
Network used for theorem 1.

We prove by showing a counter example, using the network in Figure 11. We apply our metric along with the three other techniques on the network, describe the working procedure in all these cases to show that the proposed metric indeed scores them concurrently, and finally show the difference with the other three approaches. The network in Figure 11 has 4 interactions, 2 of these are instantaneous and 2 are time-delayed (with *δ* = 1). We assume a non-trivial case where the data is supposed to be periodic (the proof is trivial otherwise). Also, we assume that all the gene expressions were discretized to 3 quantization levels. 

1. Application of MIT in a BN based framework:

(12) $$\;s_{\text{MI}T_{0}}=2\text{N.MI}(B,\{A^{0},D^{0}\})-\left(\chi_{\alpha ,4}+\chi_{\alpha ,12}\right)$$

2. Application of MIT in a DBN based framework:

(13) $$\;s_{\text{MI}T_{1}}=2N\{\text{MI}(B,C^{1})+\text{MI}(A,D^{1})\}-2\chi_{\alpha ,4}$$

3. A naive application of MIT in a combined BN and DBN based framework:

(14) $$\;\begin{array}{l} s_{\text{MI}T_{0+1}}=2N\{\text{MI}(B,\{A^{0},D^{0}\})+\text{MI}(B,C^{1})\\ \;+\text{MI}(A,D^{1})\}-\left(3\chi_{\alpha ,4}+\chi_{\alpha ,12}\right) \end{array}$$

4. Our proposed scoring metric:

(15) $$\;\begin{array}{l} s_{\text{CCIT}}=2N\{\text{MI}(B,\{A^{0},D^{0}\}\cup \{C^{1}\})\\ \;+\text{MI}(A,D^{1})\}-\left(3\chi_{\alpha ,4}+\chi_{\alpha ,12}\right)\; \end{array}$$

The concurrent scoring behavior of CCIT is evident from the first term in RHS of (15). Also, the inclusion of *C* in the parent set in the first term of the RHS of the equation exhibits the manner by which it achieves the objective of taking into account the biological fact that multiple regulators may regulate a gene jointly (the calculation, however, needs to be carried out in accordance with the process we described in the Methods Section).

Considering (12) and (13), it is also obvious that CCIT is different from both *MIT*_0_ and *MIT*_1_. To show that CCIT is different from *MIT*_0 + 1_, we consider (14) and (15). It suffices to consider whether *MI*(*B*, {*A*^0^,*D*^0^}) + *MI*(*B*, *C*^1^) is different from $\text{MI}(B,\{A^{0},D^{0}\}\cup \{C^{1}\})$. Using (11), this becomes equivalent to considering whether *MI*(*B*, {*A*^0^,*D*^0^}|*C*^1^) is the same as *MI*(*B*, {*A*^0^, *D*^0^}), which are clearly inequal. This completes the proof. □

## Endnotes

^a^The time-delay will always be greater than zero. However, if the delay is small enough so that the regulated gene is effected before the next data sample is taken, it can be considered as an instantaneous interaction

^b^a tutorial can be found in http://www.cs.ubc.ca/∼murphyk/Software/BDAGL/dbnDemo_hus.htm

^c^The method works as follows: for a variable *X*_*i*_ with *k* states, a basis vector is constructed for *P*(*X*_*i*_|*Pa*(*X*_*i*_)) by normalizing the vector $\left(\frac{1}{1},\frac{1}{2},\cdots \;,\frac{1}{k}\right)$. For the j-th instantiation *pa*(*X*_*i*_) of *Pa*(*X*_*i*_), samples are obtained for the probability corresponding to this instantiation by using *θ*_*ij*_ ∼ *Dirichlet*(*sα*_*ij*_) where *s* is the equivalent sample size and the *α*_*ij*_’s are obtained by shifting the basis vector to the right *j* places where *j* modulo *k* is not one.

^d^We clarify that different processes including genetic networks will generate scale free networks. However, if a network obtained using microarray data is scale free, it indicates that it is modelling the underlying biological process more accurately

^e^in this paper, we use Mutual Information (MI)/log-likelihood based Conditional Independence tests for analysis of regulatory interactions

^f^it should be noted here that MIT/MDL are basic scoring metric for BNs, which can be extended to score both Static and Dynamic BNs separately. Here, we are discussing MIT/MDL applied to a network having both zero and higher-order interactions

## Competing interests

The authors declare that they have no competing interests.

## Authors’ contributions

NM developed the algorithms and carried out the experiments. NM and NXV drafted the manuscript. MC and NXV suggested the biological data, experiments and provided biological insights on the results. MC provided overall supervision, direction and leadership to the research. All authors read and approved the final manuscript.

## Supplementary Material

Additional file 1 Comparison of performance with 3 other methods for the 10, 25 and 50-gene differential equation based synthetic network. Click here for file

Additional file 2 Reconstruction of SOS DNA Repair Network in *E. coli*-Experiment 2, 3, 4; results obtained using BANJO. Click here for file

Additional file 3 Reconstruction of SOS DNA Repair Network in *E. coli*-Experiment 2, 3, 4; results obtained using BNFinder+BDe. Click here for file

Additional file 4 Reconstruction of SOS DNA Repair Network in *E. coli*-Experiment 2, 3, 4; results obtained using BNFinder+MDL. Click here for file

Additional file 5 Reconstruction of SOS DNA Repair Network in *E. coli*-Experiment 2, 3, 4; results obtained using the proposed approach. Click here for file

Additional file 6 Reconstruction of SOS DNA Repair Network in *E. coli*-Experiment 2, 3, 4; results obtained using TDARACNE. Click here for file

Additional file 7 The compiled gene ontology annotation database used for the functional enrichment analysis. Click here for file
