# Supplementary material for: Simultaneous learning of instantaneous and time-delayed genetic interactions using novel information theoretic scoring technique
Source: BMC Syst Biol. 2012 Jun 12;6:62. doi: 10.1186/1752-0509-6-62 (PMC3529704; doi:10.1186/1752-0509-6-62)

Comparison of performance with 3 other methods for the 10-gene synthetic network. Red(+)-Proposed, Green(o)-Banjo, Blue(x)-BNFinder+BDe, Cyan (square)-BNFinder+MDL, Magenta(diamond)-TDARACNE

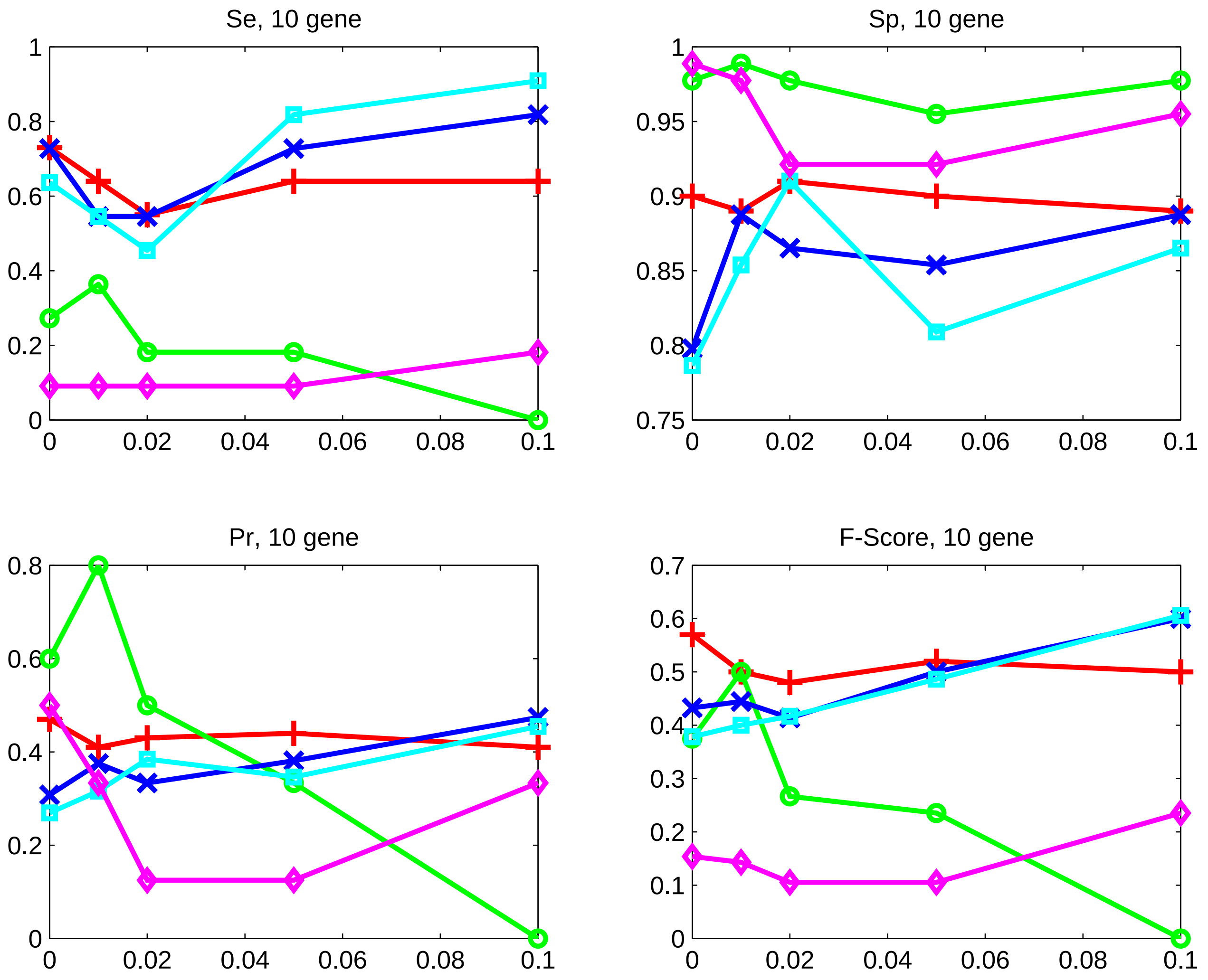

Comparison of performance with 3 other methods for the 25-gene synthetic network. Red(+)-Proposed, Green(o)-Banjo, Blue(x)-BNFinder+BDe, Cyan (square)-BNFinder+MDL, Magenta(diamond)-TDARACNE

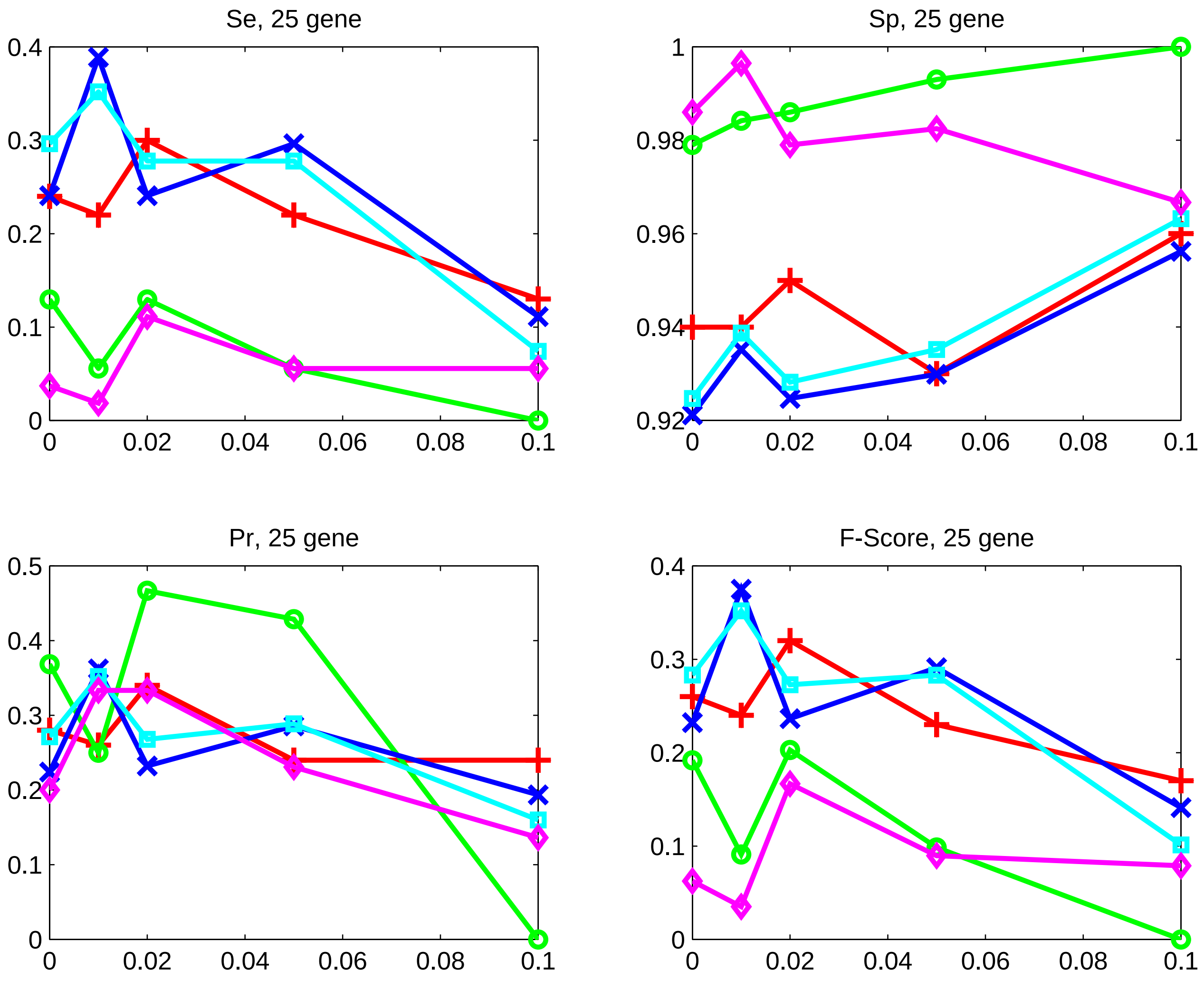

Comparison of performance with 3 other methods for the 50-gene synthetic network. Red(+)-Proposed, Green(o)-Banjo, Blue(x)-BNFinder+BDe, Cyan (square)-BNFinder+MDL, Magenta(diamond)-TDARACNE

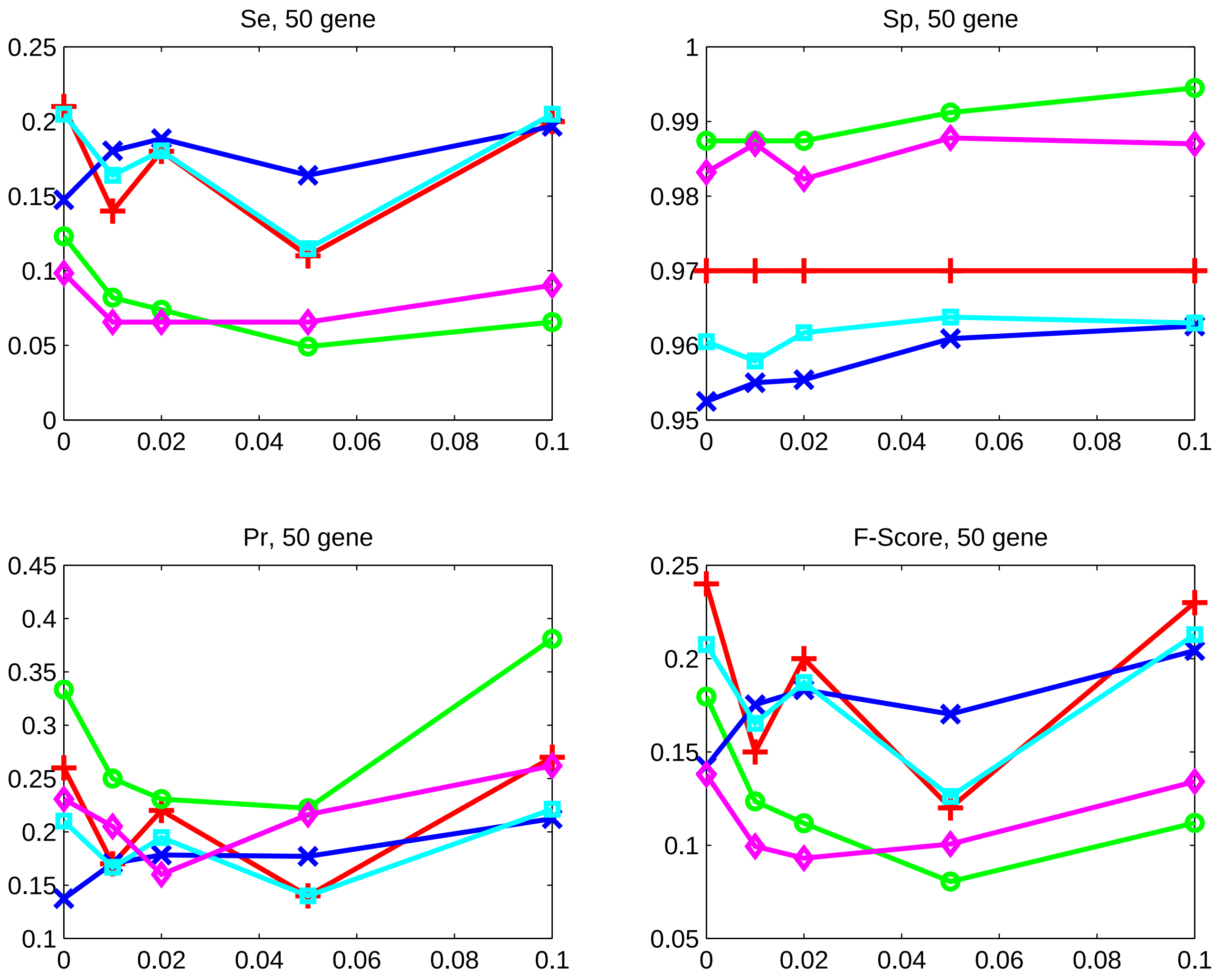

Supplement: Additional file 1 — Comparison of performance with 3 other methods for the 10, 25 and 50-gene differential equation based synthetic network. [file 1752-0509-6-62-S1.pdf]
