# Supplementary figures and images for: Simultaneous learning of instantaneous and time-delayed genetic interactions using novel information theoretic scoring technique
Source: BMC Syst Biol. 2012 Jun 12;6:62. doi: 10.1186/1752-0509-6-62 (PMC3529704; doi:10.1186/1752-0509-6-62)

Reconstruction of SOS  
DNA Repair Network in  
*E. coli*-Experiment 2,  
BNFinder+BDe

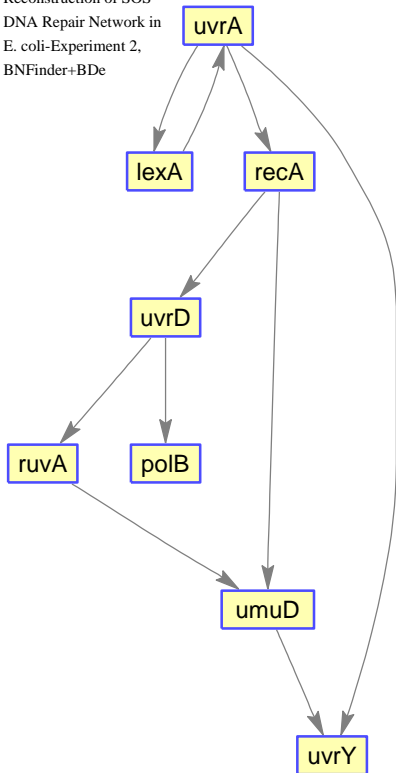

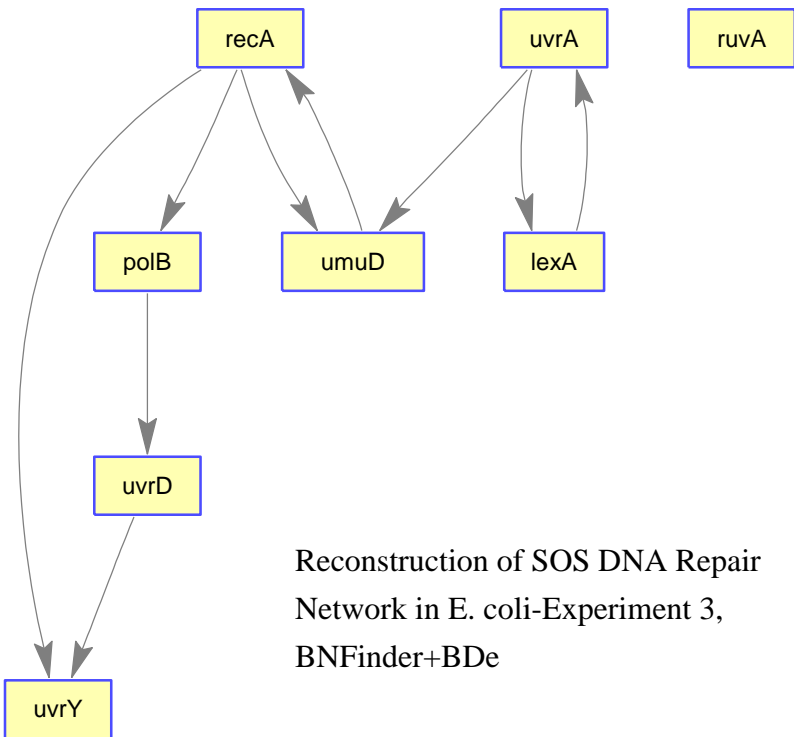

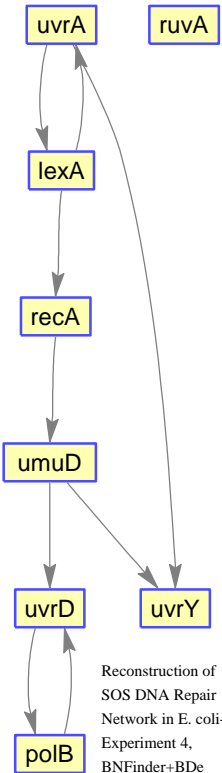

Supplement: Additional file 3 — Reconstruction of SOS DNA Repair Network in E. coli-Experiment 2, 3, 4; results obtained using BNFinder+BDe. [file 1752-0509-6-62-S3.pdf]
