# Supplementary material for: Simultaneous learning of instantaneous and time-delayed genetic interactions using novel information theoretic scoring technique
Source: BMC Syst Biol. 2012 Jun 12;6:62. doi: 10.1186/1752-0509-6-62 (PMC3529704; doi:10.1186/1752-0509-6-62)
Supplement: Additional file 6 — Reconstruction of SOS DNA Repair Network in E. coli-Experiment 2, 3, 4; results obtained using TDARACNE. [file 1752-0509-6-62-S6.pdf]

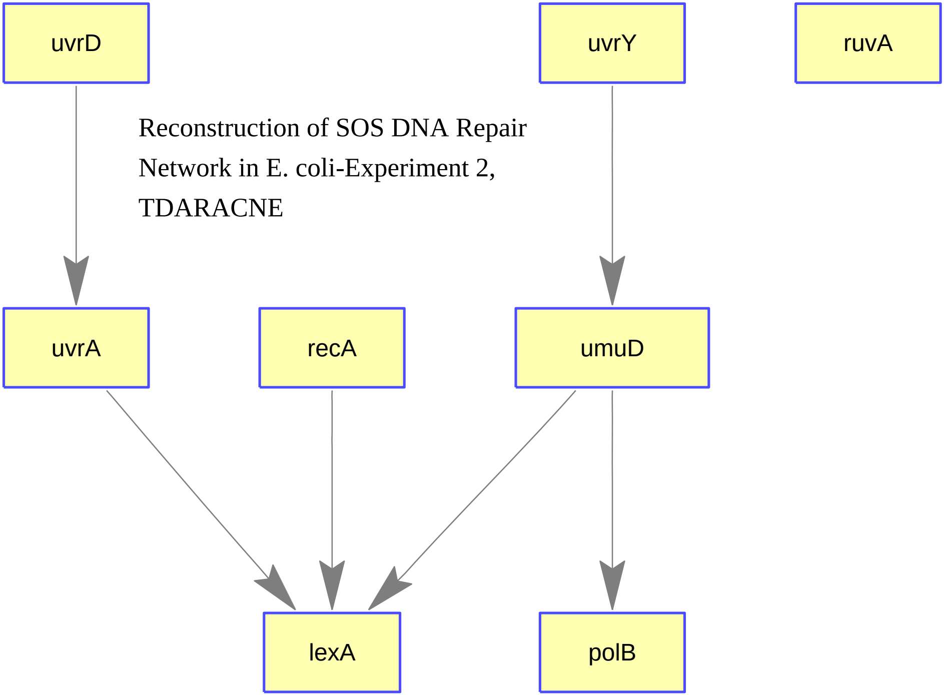

Reconstruction of SOS DNA Repair Network in E. coli-Experiment 2, TDARACNE

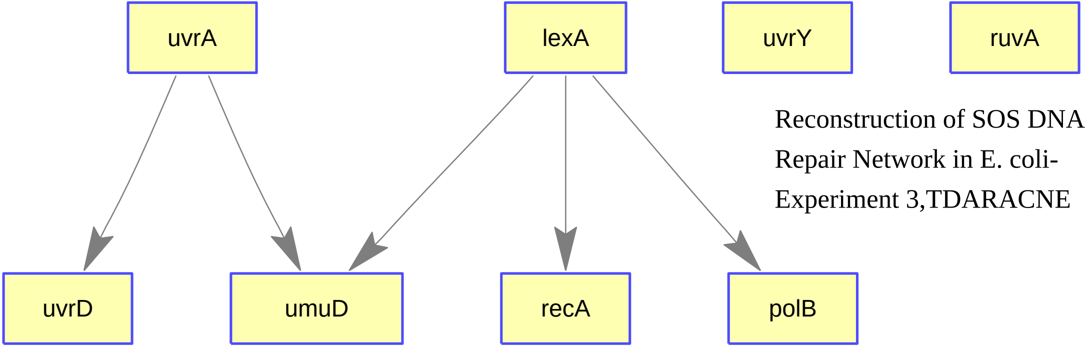

Reconstruction of SOS DNA Repair Network in E. coli- Experiment 3,TDARACNE

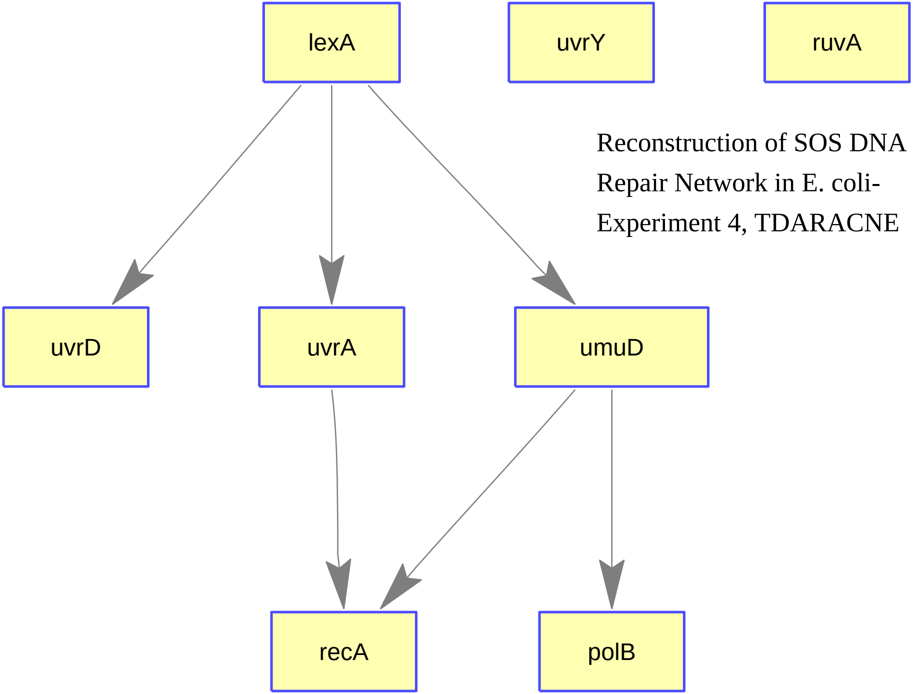

Reconstruction of SOS DNA Repair Network in E. coli- Experiment 4, TDARACNE
